# RBFOX1 and RBFOX2 are dispensable in iPSCs and iPSC-derived neurons and do not contribute to neural-specific paternal *UBE3A* silencing

**DOI:** 10.1038/srep25368

**Published:** 2016-05-05

**Authors:** Pin-Fang Chen, Jack S. Hsiao, Carissa L. Sirois, Stormy J. Chamberlain

**Affiliations:** 1 Department of Genetics and Genome Sciences, University of Connecticut Health Center, Farmington, Connecticut, 06030, USA; 2 Department of Neuroscience, University of Connecticut Health Center, Farmington, Connecticut, 06030, USA; 3 University of Connecticut Stem Cell Institute, University of Connecticut Health Center, Farmington, Connecticut, 06030, USA

## Abstract

Angelman Syndrome (AS) is a rare neurodevelopmental disorder caused by loss of function of the maternally inherited copy of *UBE3A*, an imprinted gene expressed biallelically in most tissues, but expressed exclusively from the maternal allele in neurons. Active transcription of the neuron-specific long non-coding RNA (lncRNA), *UBE3A-ATS,* has been shown to silence paternal *UBE3A*. We hypothesized that alternative splicing factors RBFOX2 and RBFOX1 might mediate splicing changes and result in the transcription of *UBE3A-ATS* in neurons. We found that RBFOX2 and RBFOX1 both bind to *UBE3A-ATS* transcript in neurons, but are not required for gene expression and/or neuron-specific processing in the *SNURF/SNRPN-UBE3A* region. However, we found that depletion of RBFOX2 causes a proliferation phenotype in immature neural cultures, suggesting that RBFOX2 is involved in division versus differentiation decisions in iPSC-derived neural progenitors. Absence of RBFOX2 also altered the expression of some genes that are important for glutamatergic neocortical development and Wnt-Frizzled signalling in mature neuronal cultures. Our data show that while RBFOX1 and RBFOX2 do not mediate neuron-specific processing of *UBE3A-ATS*, these proteins play important roles in developing neurons and are not completely functionally redundant.

Angelman syndrome (AS), a neurodevelopmental disorder affecting approximately 1/15,000–1/30,000 live births^1^, is characterized by microcephaly, seizures, ataxia, lack of speech, happy demeanor, and severe cognitive disability^2^. AS is caused by the loss of function from the maternal copy of the *UBIQUITIN PROTEIN LIGASE E3A* (*UBE3A*) gene^3^. *UBE3A* is imprinted, and expressed preferentially from the maternal allele in neurons, but is expressed from both parental alleles in other tissues. This tissue-specific imprinting—or silencing of paternal *UBE3A*—occurs due to the neuron-specific expression of an antisense transcript (*UBE3A-ATS*) from the paternal allele^4^. *UBE3A-ATS* is one of several lncRNAs that are processed from the *SNURF/SNRPN* gene. In addition to the protein-coding *SNURF* and *SNRPN* transcripts, this gene also produces several members of the *SNORD116* and *SNORD115* snoRNA clusters, as well as lncRNAs of unknown function, such as *IMPRINTED IN PRADER-WILLI* (*IPW*) and *UBE3A-ATS*^5^. The neuron-specific regulation of *UBE3A-ATS* is poorly understood, but likely differs between mouse and human. In mouse, the entire lncRNA portions of the *Snurf/Snrpn* transcript are neuron-specific^6^. In human, the lncRNAs between *SNURF/SNRPN* protein-coding portion and the *IPW* lncRNA are expressed broadly in many tissue types, but the lncRNA downstream of *IPW*, including the *SNORD115* cluster and *UBE3A-ATS* are neuron-specific^4^. It is important to understand the regulation of the neuron-specific portion of *SNURF/SNRPN* lncRNA, since it, in turn, controls *UBE3A* imprinted expression^7^,^8^.

A previous crosslinking-immunoprecipitation-sequencing (CLIP-Seq) study in human embryonic stem cells (hESCs) showed that the *SNURF/SNRPN* lncRNA is highly bound by an alternative splicing factor, RBFOX2 (data available at http://genome.ucsc.edu)^9^. RBFOX2, also known as RBM9, belongs to the RBFOX1 family of RNA-binding proteins^10^. RBFOX2 and its paralogs, RBFOX1 (A2BP1) and RBFOX3 (HRNBP3), are known to be key regulators of alternative splicing in neurons and regulate exon exclusion/inclusion as well as intron retention^10^,^11^,^12^. All three RBFOX proteins are known to bind to the hexanucleotide sequence UGCAUG, a common *cis*-regulatory element in pre-mRNAs. Mouse studies suggest that RBFOX1 is highly expressed in heart, brain, and muscles while RBFOX2 is expressed in a broader range of tissues, including the aforementioned as well as ovary, kidney, lung epithelium, and embryo^11^. In comparison, RBFOX3 is not as well characterized and is identified to encode a neuron-specific nuclear protein that is recognized by the NeuN antibody^13^. Recent studies indicate that RBFOX proteins may be involved in alternative polyadenylation regulation and affect functions related to 3′ untranslated regions (UTRs), such as mRNA stability, localization, and translation^14^,^15^. Interestingly, RBFOX1 haploinsufficiency is linked to autism, intellectual disability, and epilepsy^16^.

High-throughput sequencing studies of 3′ polyadenylated ends (polyA-Seq) in human tissues revealed two polyadenylation sites at the 3′ end of *IPW* near the end of the *SNURF/SNRPN* lncRNA transcript in non-neuronal cells^17^. We hypothesized that the neuron-specific expression of RBFOX1 regulates an alternative splicing event that results in the skipping of the polyadenylation sites at *IPW*, leading to the neuron-specific expression of *UBE3A-ATS* and subsequent imprinted expression of *UBE3A*. In this paper, we investigated whether depletion of RBFOX1 and RBFOX2 altered *SNURF/SNRPN* lncRNA expression and/or processing during neural differentiation. We mutated RBFOX1 and RBFOX2 singly and in combination using lentiCRISPRs in AS and normal patient-derived iPSCs and differentiated them into neurons. We found that RBFOX1 and RBFOX2 are not required for the neuron-specific expression or processing of the *SNURF/SNRPN*, even though this is one of the most highly bound RNAs by RBFOX2 in pluripotent stem cells. However, we found a neural differentiation defect in RBFOX2-null iPSCs, suggesting that RBFOX2 is involved in division versus differentiation decisions in iPSC-derived neural progenitors.

## Results

### RBFOX2 is expressed ubiquitously throughout *in vitro* neural differentiation while RBFOX1 is expressed in a neuron-specific manner in human

**Figure 1:**
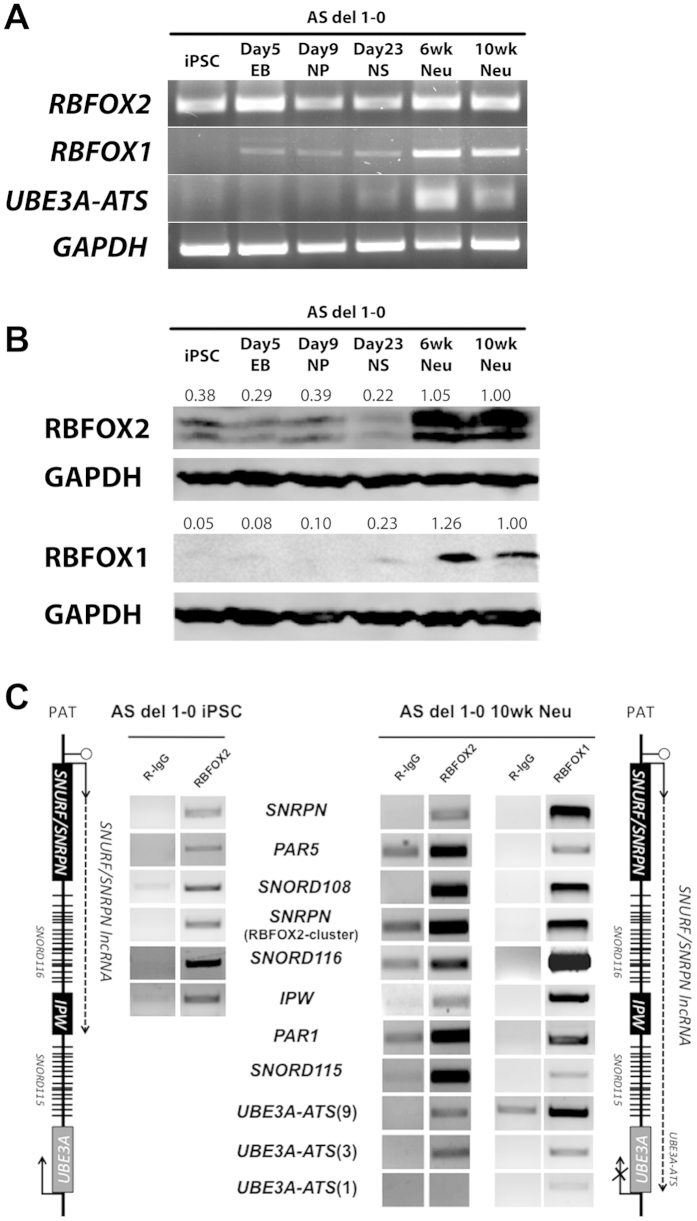
RBFOX expression and binding during neural differentiation (iPSC: induced pluripotent stem cell; EB: embryoid body; NP: neural precursor; NS: neural sphere; Neu: neuron). (**A**) Conventional RT-PCR showing RNA expression of *RBFOX2*, *RBFOX1*, *UBE3A-ATS*, and *GAPDH* over 10 weeks of *in vitro* neural differentiation. (**B**) Western blots showing protein expression of RBFOX2 and RBFOX1. GAPDH was used as a loading control. Numbers indicate GAPDH-normalized protein levels relative to 10-week-old neurons. (**C**) RBFOX2 and RBFOX1 bind to *SNURF/SNRPN* lncRNA in iPSCs and 10-week-old neurons. Rabbit IgG served as a negative control for non-specific binding.

Previous studies indicated that *RBFOX2* and its protein product are expressed in a broad spectrum of tissues and cell types, including hESCs^9^,^11^, while *RBFOX1* and its protein product are specifically expressed in brain and muscle cells^11^. We sought to determine the expression patterns of RBFOX2 and RBFOX1 RNA and protein during *in vitro* differentiation of human iPSCs into neurons. Conventional PCR and western blotting in iPSCs and their neural-derivatives revealed that RBFOX2 is expressed abundantly in iPSCs and at all time points during neural differentiation (Fig. 1A,B). On the other hand, *RBFOX1* RNA is first evident in the neural precursor stage, but is markedly upregulated in 6-week and 10-week neuronal cultures (Fig. 1A). RBFOX1 protein is detectable only in neural cultures that have been maturing *in vitro* for over 6 weeks (Fig. 1B). Robust expression of *RBFOX1* RNA and detectable expression of RBFOX1 protein is coincident with the appearance of *UBE3A-ATS* (Fig. 1A)^4^. A third paralog, RBFOX3, is first detectable in 10-week neuronal cultures, weeks after the initial detection of *UBE3A-ATS* (Supplementary Figure 7).

### *SNURF/SNRPN* lncRNA transcripts are bound by RBFOX1 and RBFOX2 in iPSCs and neurons

Since RBFOX1 and RBFOX2 are both RNA-binding proteins^18^ and RBFOX2 was previously shown to bind the *SNURF/SNRPN* lncRNA, we carried out cross-linking immunoprecipitation (CLIP) to see whether the RBFOX proteins bind *SNURF/SNRPN* in iPSCs and 10-week-old iPSC-derived neurons (Fig. 1C). We found abundant RBFOX2-binding on the *SNURF/SNRPN* lncRNA expressed in iPSCs (i.e. from *SNURF/SNRPN* to *IPW*). This finding agrees with previously published RBFOX2 CLIP-seq data in hESCs^9^. In neurons, where both RBFOX1 and RBFOX2 are expressed, we found both factors bound to the entire *SNURF/SNRPN* lncRNA, including neuron-specific *SNORD115* and *UBE3A-ATS*. Since RBFOX1 expression correlated with the appearance of *UBE3A-ATS,* and since RBFOX proteins bind to *SNURF/SNRPN* lncRNA transcripts, we hypothesized that RBFOX1, by itself or together with RBFOX2, may play a role in regulating the expression of the neuron-specific portion of *SNURF/SNRPN* transcripts via alternative splicing.

### Loss of RBFOX2 does not affect expression of the lncRNA in iPSCs and neurons

**Figure 2:**
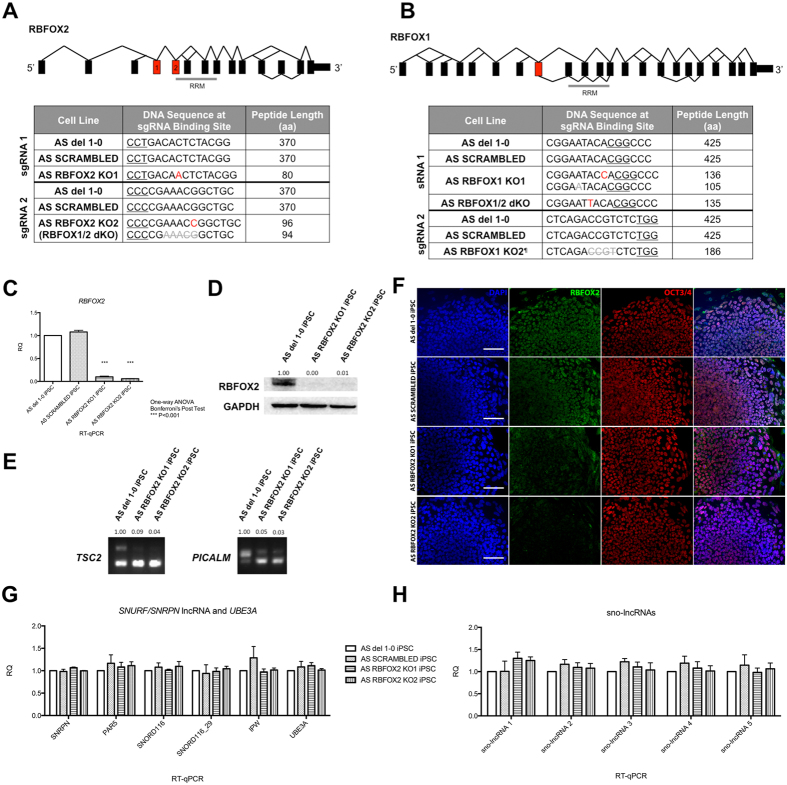
Lentiviral CRISPR/Cas9-mediated RBFOX KO in AS iPSCs. (**A,B**) Schematic of *RBFOX2* and *RBFOX1* splicing patterns according to the alternative splicing graph from Swiss Institute of Bioinformatics on UCSC genome browser. Targeted exonss are colored in red and the numbers correspond to respective sgRNAs. gDNA sequencing shows frameshift indels leading to premature stop codon in two RBFOX2 KOs, two RBFOX1 KOs, and one RBFOX1/2 dKO (^¶^see Supplemental Figure 2). (**C**) RT-qPCR, (**D**) western blot (numbers indicate GAPDH-normalized protein levels relative to un-manipulated AS del 1-0 iPSCs), and **(F**) immunocytochemistry showing RBFOX2 mRNA or protein expression in RBFOX2 KO iPSCs. Scale bar, 100 μm. (**E**) Splicing changes in *TSC2* and *PICALM* showing functional loss of RBFOX2. Numbers indicate exon-inclusion over exon-exclusion events relative to un-manipulated AS del 1–0 iPSCs. (**G,H**) The expression of transcripts from *SNURF/SNRPN* lncRNA, including *SNORD116* and *sno-lncRNAs*, was not altered in RBFOX2 KO iPSCs.

To investigate the role of RBFOX2 in the regulation of *SNURF/SNRPN* lncRNA, we knocked out RBFOX2 in AS iPSCs harboring a large deletion of maternal 15q11-q13 using CRISPR/Cas9. Specifically, we transduced AS iPSCs with lentiviruses carrying both Cas9 and sgRNA components^19^ to create non-homologous end-joining (NHEJ)-mediated insertions/deletions (indels). To account for potential off-target effects, we designed two different sgRNAs targeting the two most upstream exons of *RBFOX2* that are common amongst all *RBFOX2* transcripts (Fig. 2A). As a control for lentiviral transduction and CRISPR/Cas9 integration and expression, we designed a scrambled sgRNA that has no match in the human genome^20^. For RBFOX2 knockouts (KOs), we amplified and sequenced the genomic DNA near the predicted CRISPR/Cas9 cut site to identify small indels that shifted the reading frame, leading to a premature stop codon. We selected two clonal iPSC lines for further study.

We then quantified *RBFOX2* mRNA and protein and found reduced levels of *RBFOX2* mRNA (Fig. 2C) and undetectable levels of RBFOX2 protein in RBFOX2 KO iPSCs (Fig. 2D,F). We also confirmed the functional loss of RBFOX2 by assaying splicing changes in previously reported RBFOX2-splicing targets, *PICALM* and *TSC2* (Fig. 2E)^9^. Even though RBFOX2 is the only available RBFOX paralog expressed in iPSCs, the absence of RBFOX2 did not affect gene expression at any portion of the *SNURF/SNRPN* lncRNA assayed (Fig. 2G,H). Specifically, we quantified *SNORD116* host transcript and *sno-lncRNAs*, which are most enriched for RBFOX2 binding^9^,^21^, as well as an individual processed snoRNA, *SNORD116–29*, and found no expression differences between non-transduced, scrambled and RBFOX2 KO iPSCs.

**Figure 3:**
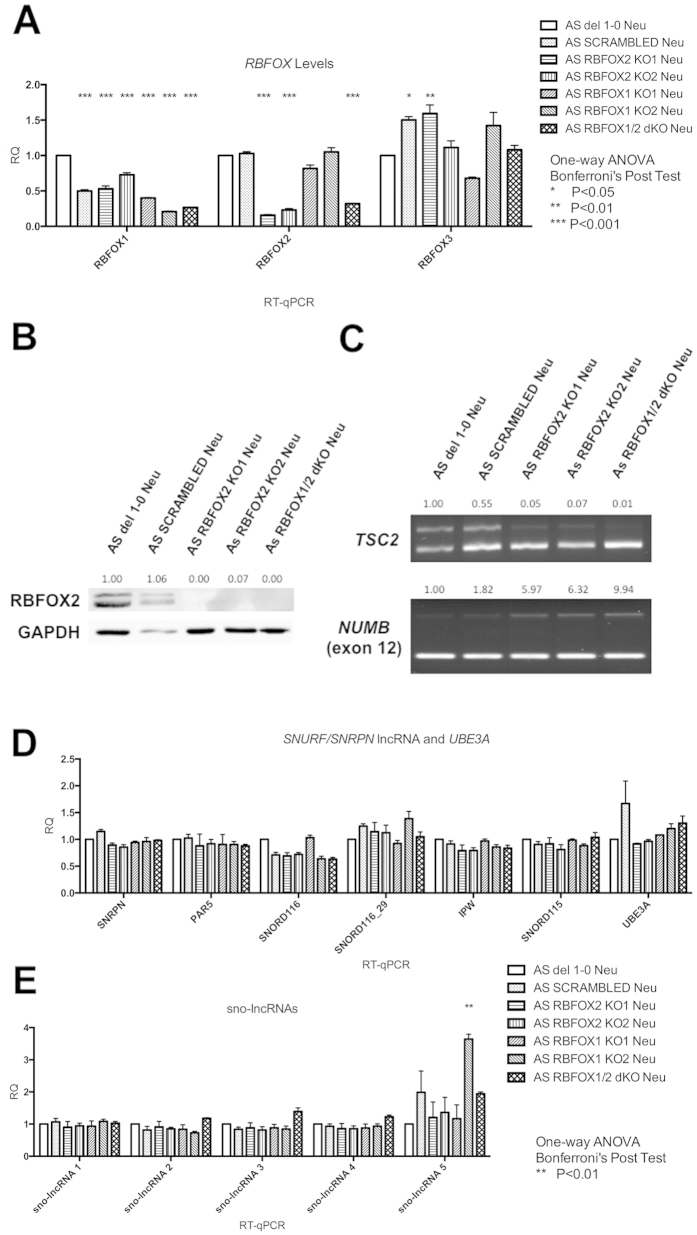
Expression of transcripts from *SNURF/SNRPN* lncRNA was not altered in RBFOX KO AS iPSC-derived neurons. (**A**) RT-qPCR showing *RBFOX2* and *RBFOX1* expression in respective KO neurons. (**B**) Western blot of RBFOX2 protein in KO neurons. Numbers indicate GAPDH-normalized protein levels relative to un-manipulated AS del 1–0 neurons. Uneven loading led to decreased band intensities in scrambled control neurons for both RBFOX2 and GAPDH. (**C**) Splicing changes in *TSC2* and *NUMB* in RBFOX2 KO neurons. Numbers indicate exon-inclusion over exon-exclusion events relative to un-manipulated AS del 1–0 neurons. (**D,E**) Expression of transcripts from *SNURF/SNRPN* lncRNA, including *SNORD116* and *sno-lncRNA,* in RBFOX KO neurons.

Following 10-week *in vitro* neural differentiation, we reassessed *RBFOX2* mRNA and protein levels, and ensured that functional loss of RBFOX2 was still evident in RBFOX2 KO neurons (Fig. 3A–C). Specifically, we observed decreased inclusion of a 129nt exon of *TSC2* and increased inclusion of exon 12 of *NUMB* in RBFOX2 KO neurons. Since it is known that RBFOX paralogs affect each other’s splicing and expression, we also quantified the RNA levels of *RBFOX1* and *RBFOX3*, which are expressed in neurons. We observed a slight downregulation of *RBFOX1* and upregulation in *RBFOX3* in RBFOX2 KO neurons. However, these changes were also observed in the scrambled control (Fig. 3A), and are thus unlikely to be related to RBFOX2 depletion. As in iPSCs, the absence of RBFOX2 did not overtly affect the expression of the *SNURF/SNRPN* lncRNA (Fig. 3D,E).

To rule out the possibility that RBFOX2 might play a different role in normal iPSCs and neurons, we also mutated RBFOX2 in normal iPSCs and differentiated them into neurons (Supplementary Figure 1). Although we found slight increases in the levels of *RBFOX1* and *RBFOX3* mRNAs as well as small, but significant increases in 4 out of 5 *sno-lncRNAs* in the RBFOX2 KO neurons, we determined that the RBFOX2 KO neurons also had increased *MAP2* and *VGLUT2* levels, suggesting that there are more mature neurons in the RBFOX2 KO cultures. This variability in the neuronal cultures may account for the increases in *RBFOX1* and *RBFOX3* mRNAs and the *sno-lncRNAs* independent of RBFOX2 depletion (Supplementary Figure 1I).

### Loss of RBFOX1 does not affect the expression of the *SNURF/SNRPN lncRNA* in neurons

We hypothesized that tissue-specific expression of RBFOX1 might play a critical role in processing the *SNURF/SNRPN* lncRNA, leading to the neuron-specific expression of *UBE3A-ATS* and repression of paternal *UBE3A*. Therefore, we knocked out RBFOX1 in AS iPSCs and in RBFOX2 KO AS iPSCs using the lentiCRISPR/Cas9 approach. The details of the specific mutations further studied are shown in Fig. 2B. Following differentiation into 10-week neurons, we found that *RBFOX1* mRNA was significantly reduced in RBFOX1 KO and RBFOX1/2 double knockout (dKO) iPSC-derived neurons (Fig. 3A). However, *RBFOX1* was reduced in RBFOX2 KO neurons, and neurons derived from iPSCs transduced with the scrambled lentiCRISPR as well. This is most likely due to variability in neural differentiation, although we cannot rule out the possibility that the scrambled lentiCRISPR or the insertional mutations caused by its integration into the genome reduces *RBFOX1* or affects neural differentiation. We were not able to obtain the same lot of RBFOX1 antibody that had worked for Western blot previously. Therefore, to assure the absence of functional RBFOX1 protein, we cloned cDNAs from the RBFOX1 KO and RBFOX1/2 dKO neurons to ensure that the remaining RNA transcripts harbored frame-shift mutations and lead to premature stop codons (Supplementary Figure 2). Unexpectedly, in RBFOX1 KO2, we found a subpopulation of neurons expressing *RBFOX1* cDNA with a complex 57nt deletion that was not identified in the original iPSC gDNA. By designing primers specific to the unique DNA sequence created by this deletion, we found a very small population of iPSCs presenting the same mutation (Supplementary Figure 2F). This indicates that RBFOX1 KO2 was a mixed clone, with the majority harboring a 4nt frame-shift deletion in *RBFOX1*. The functional loss of RBFOX1 in neurons was confirmed by splicing changes in previously reported RBFOX1 targets, *KCND3* and *ABLIM1* (Supplementary Figure 2G)^22^. To our surprise, the expression of the *SNURF/SNRPN* lncRNA and *UBE3A* was not significantly affected in RBFOX1 KO and RBFOX1/2 dKO neurons (Fig. 3D,E).

### RBFOX2 KO iPSCs are viable with normal cell cycle and have no increase in apoptosis

**Figure 4:**
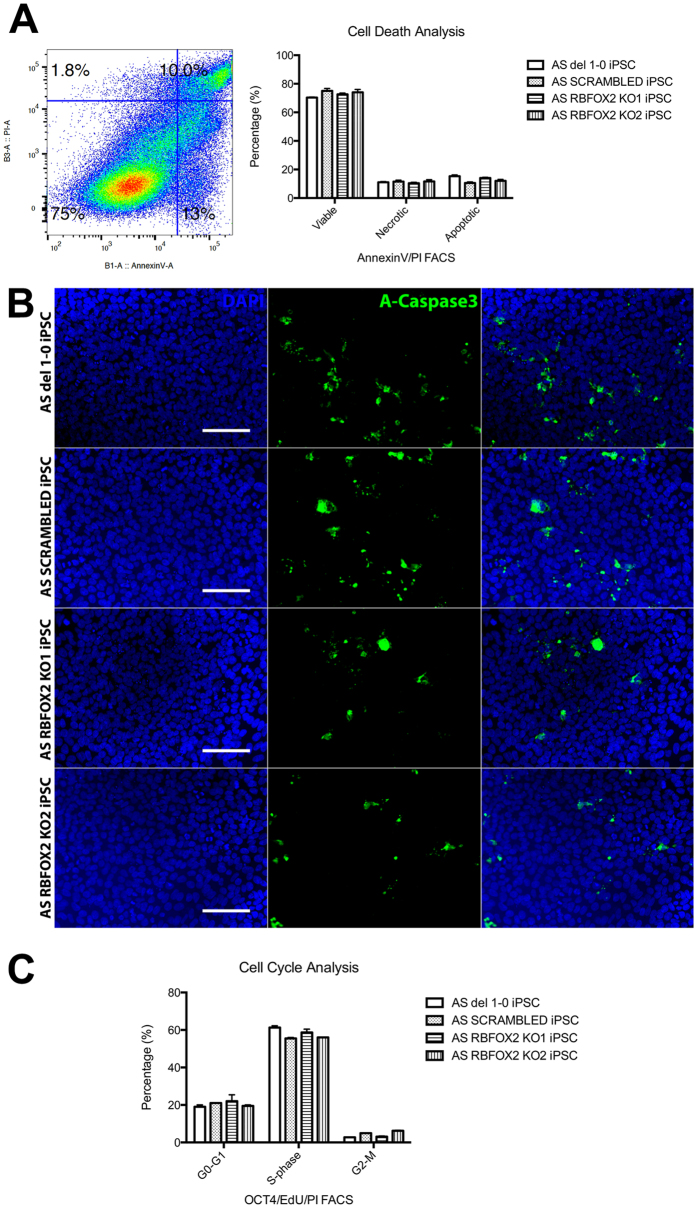
Cell death and cell cycle analysis in RBFOX2 KO AS iPSCs. (**A**) Flow cytometry analysis using Annexin V and propidium iodide, to identify viable, necrotic, or apoptotic cells. (**B**) Apoptotic cells labeled with activated caspase-3 (green). Scale bar, 100 μm. (**C**) Flow cytometry was used to analyse cell cycle in synchronized iPSCs. Cells were labeled with EdU during S-phase, and then stained with propidium iodide.

Contrary to Yeo *et al.*^9^, we found that RBFOX2 KO iPSCs in both AS and normal background were viable and did not exhibit increased apoptosis (Fig. 4A, Supplementary Figure 3A). To confirm this finding, we performed immunocytochemistry using an antibody against activated-caspase3 and found no significant differences between the controls and RBFOX2 KO iPSCs (Fig. 4B, Supplementary Figure 3B). We also tested whether the absence of RBFOX2 affects cell cycle. RBFOX2 KO and control iPSCs were synchronized using a nocodazole block and labeled using EdU. We found no differences between RBFOX2 KO iPSCs and controls at any stage of the cell cycle (Fig. 4C, Supplementary Figure 3C). This indicated that RBFOX2 KO iPSCs also have a normal cell cycle length.

### RBFOX2 KOs exhibit increased proliferation during *in vitro* neural differentiation

**Figure 5:**
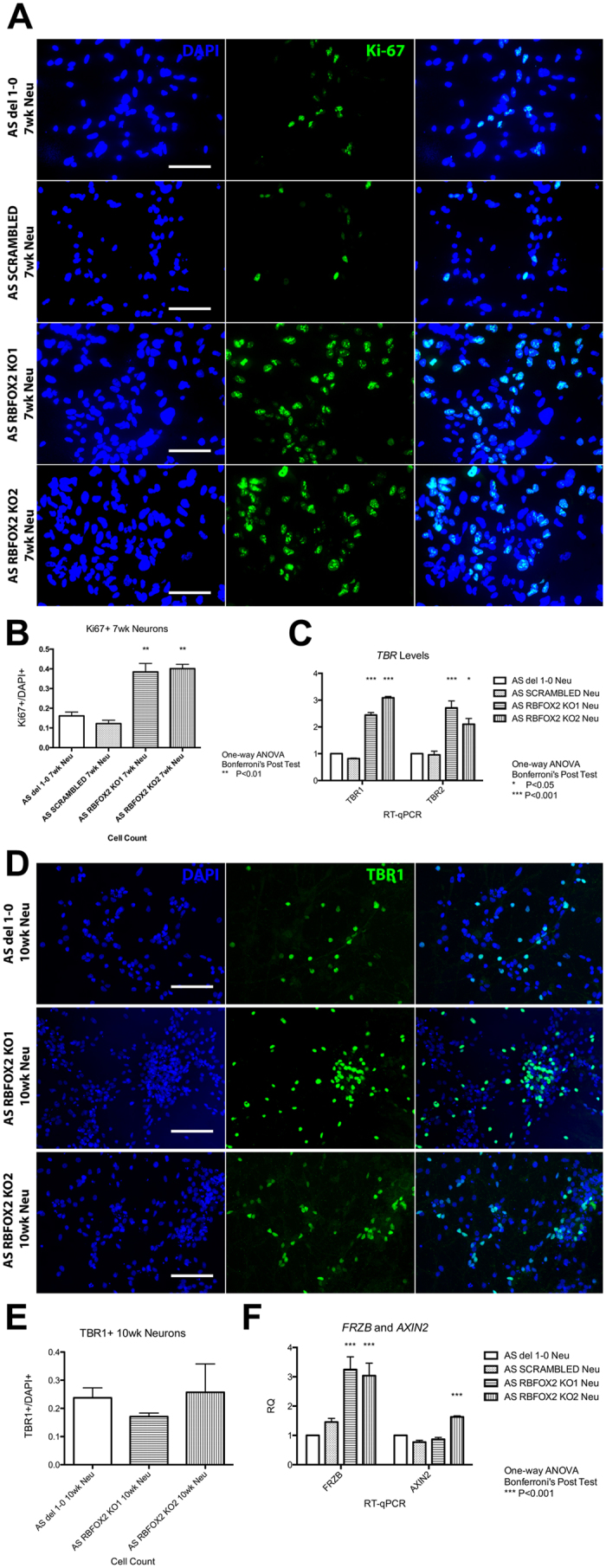
7-week-old AS RBFOX2 KO neurons showed increased proliferation and 10-week-old AS RBFOX2 KO neurons showed increased *TBR1*, *TBR2*, and *FRZB* expression. (**A**) Proliferating cells in 7-week-old neural culture labeled with antibody against Ki67 (green). Scale bar, 100 μm. (**B**) Quantification of Ki67 + cells in A. (**C**) RT-qPCR showing *TBR1* and *TBR2* expression in 10-week-old RBFOX2 KO neurons. (**D**) 10-week-old RBFOX2 KO neurons labeled with antibody against TBR1 (green). Scale bar, 100 μm. (**E**) Quantification of TBR1 + cells in Figure 5D (**F**) RT-qPCR showing *FRZB* and *AXIN2* expression in 10-week-old RBFOX2 KO neurons.

Although the iPSCs appeared to be normal, we consistently saw increased proliferation of RBFOX2 KO neurons in all three RBFOX2 KO lines—two on AS background and one on normal background—at approximately 7 weeks of differentiation when compared to their respective scrambled and unmanipulated controls. Immunocytochemistry using an antibody against Ki67 confirmed that the proliferating population is significantly increased in RBFOX2 KO 7-week neuronal cultures when compared to controls (Fig. 5A,B, Supplementary Figure 4A,B). This proliferation phenotype in RBFOX2 KO neurons could be caused by splicing changes in exon 12 of *NUMB* (Fig. 3C). Exon 12 alternative splicing in NUMB is mediated by RBFOX2^23^, the inclusion of which is pro-proliferation while the exclusion has been shown to be pro-differentiation during neural development^24^.

To determine whether this increase in proliferation affects neural cell-fate and, hence, the cell population in the 10-week culture, we quantified mRNA levels of neural markers in 10-week-old AS neurons (Supplemental Figure 5). Markers representing neuronal precursors (*SOX2*, *PAX6*, and *DCX*), neurons (*TUBB3* and *CTIP2*), and mature astrocytes (*S100B*) were largely unchanged in RBFOX2 KO neurons. However, significant increases in *TBR1* and *TBR2* were observed in RBFOX2 KO neurons (Fig. 5C, Supplementary Figure 4C). *TBR2* is a marker of intermediate progenitor cells, while *TBR1* is a marker for post-mitotic cortical plate neurons^25^. By counting the number of cells that express TBR1 in Fig. 5D, we found that the percentage of TBR1 positive cells in the neuronal culture was comparable between the controls and RBFOX2 KO neurons (Fig. 5E, Supplementary Figure 4D,E), suggesting that TBR1 may be upregulated in individual cells and that the proportion of post-mitotic cortical plate neurons was not increased.

### *FRZB* mRNA is upregulated in RBFOX2

Gene expression changes were previously seen in RBFOX1 knockdown human neural progenitor cells^26^. We sought to determine whether these changes could also be seen in RBFOX1 and RBFOX2 KO neurons. We observed no significant expression changes in *SV2B*, *NRXN1*, *GABRA3*, and *FRZB* in AS RBFOX1 KO and RBFOX1/2 dKO neurons compared to the controls (Supplementary Fig. 6). However, we observed an increase in *FRZB* expression in normal and AS RBFOX2 KO neurons (Fig. 5F, Supplementary Figure 4F). FRZB protein competes with FRIZZLED receptors for Wnt-binding^27^,^28^. Increased *FRZB* levels suggest that Wnt/β-catenin signaling might be attenuated in RBFOX2 KO neurons. Therefore, we quantified *AXIN2* expression, which is transcriptionally activated by Wnt/β-cat signaling^29^. Unexpectedly, we saw a subtle increase *AXIN2* expression, rather than the expected decrease (Fig. 5F, Supplementary Figure 4F). These data suggest that although increased *FRZB* mRNA is observed in RBFOX2 KO neurons, there is no evidence of attenuated Wnt/β-catenin signaling.

## Discussion

RBFOX2 and its paralog, RBFOX1 are RNA binding proteins known to regulate alternative splicing in brain^22^,^30^. Yeo *et al.* showed abundant RBFOX2-binding on the *SNURF/SNRPN* lncRNA in hESCs^9^. We hypothesized that RBFOX1, the neuron-specific paralog of RBFOX2, may regulate alternative splicing of the *SNURF/SNRPN* lncRNA, leading to the skipping of the polyadenylation sites in *IPW* and neuron-specific expression of the distal portion of the lncRNA, also known as *UBE3A-ATS*. We first determined that expression of the RBFOX1 protein is coincident with the appearance of *UBE3A-ATS*, while RBFOX2 protein is more broadly expressed. A third paralog, RBFOX3, is only expressed in 10-week neuronal cultures, weeks after the initial detection of *UBE3A-ATS* (Supplementary Figure 7). Because RBFOX3 was expressed at a time point later than the first appearance of *UBE3A-ATS*, it is unlikely to play a role in regulating *UBE3A-ATS* expression and was not considered further in these studies, although it is formally possible that RBFOX3 is upregulated in the absence of RBFOX1 and/or RBFOX2. Brainspan data support these observations (www.brainspan.org)^31^,^32^. RBFOX2 is abundantly expressed in the earliest stages of human brain development, while RBFOX1 becomes reliably expressed beginning at 11 weeks post-conception. RBFOX3 is upregulated even later in neuronal development (Supplementary Figure 7). Subsequently, we found abundant RBFOX2 and RBFOX1 binding across the entire *SNURF/SNRPN* lncRNA in human iPSC-derived neurons, including *IPW* and *UBE3A-ATS*. These observations supported our initial hypothesis that RBFOX1, by itself or together with RBFOX2, may play a role in regulating the expression of the neuron-specific portion of *SNURF/SNRPN* transcripts via alternative splicing.

To directly test whether RBFOX1 and/or RBFOX2 played a role in the neuron-specific processing of the *SNURF/SNRPN* lncRNA, we knocked out RBFOX2 and RBFOX1 alone and in combination in AS iPSCs. These cells have a deletion of the maternal allele of chromosome 15q11-q13, allowing us to focus on the RNA transcripts originating from the paternal allele. We used lentiCRISPR/Cas9 technology to generate indels that would create a frameshift and prematurely stop RBFOX protein translation. Although puromycin-resistant clones were carefully screened manually and using online TIDE (Tracking of Indels by Decomposition) software^33^ to ensure that they were clonal populations with the desired deleterious mutations, we found that at least one clone harbored a mutation larger than 50 nucleotides, which was not readily detectable by TIDE or manual efforts. The RBFOX1 KO2 clone carried a small portion of cells with a 57 nt in-frame deletion, which likely produces a small amount of potentially functional RBFOX1 protein (Supplementary Figure 2). All other clones were demonstrated to have either loss of RBFOX protein and/or showed only the presence of a mutated cDNA that is predicted to make a prematurely terminated protein (Supplementary Figure 2).

Upon knocking out RBFOX2 and RBFOX1, individually and in combination, we found that *SNURF/SNRPN* lncRNA expression levels, including the steady-state levels of the *SNORD116* host gene, an individual processed *SNORD116* snoRNA, the *sno-lncRNAs*, and the *SNORD115* host gene were not altered in iPSCs and in neurons. This indicates that even though RBFOX2 and RBFOX1 bind to *SNURF/SNRPN* lncRNA, they are not essential for its processing and neuron-specific expression as we hypothesized. This is surprising since the *SNURF/SNRPN* lncRNA represents one of the strongest binding sites for RBFOX2 in human pluripotent stem cells^9^, and since RBFOX1 is known to play and important role in normal human brain development^16^. In addition, we found that the absence of RBFOX2 did not change *SNORD116* localization in the nucleus (Supplementary Figure 8), indicating that RBFOX2 does not have an obvious role in in nuclear organization of this locus. The unaltered steady-state levels of *SNORD115* RNA in RBFOX1KO and RBFOX1/2 dKO neurons suggest that RBFOX1 is not regulating alternative polyadenylation as we had originally hypothesized. It is possible that alternative polyadenylation is regulated by a different RNA binding protein, or alternatively, the *SNURF/SNRPN* lncRNA is truncated in non-neurons by a different mechanism.

In agreement with our results, Yin *et al.* reported that RBFOX2 knockdown did not affect *sno-lncRNA* levels in PA1 cells^21^, and further hypothesized that RBFOX proteins may be sequestered by the *lncRNA*-binding. Our data did not address the latter notion. Yeo *et al.* reported a positive correlation between the amount of RBFOX2 binding and transcript abundance^9^, thus, the enrichment for RBFOX binding on *SNURF/SNRPN* lncRNA and its processed products may reflect the abundance of these RNA transcripts. We did not observe increased cell death in RBFOX2 knockout iPSCs as previously reported in RBFOX2 knockdown hESCs^9^. The disparity in viability may be due to differences between RBFOX2 knockdown versus knockout, but is most likely either caused by differences in the culture system (i.e. feeder-free versus MEF feeders) for the pluripotent stem cells or shRNA toxicity.

Despite the fact that many important neuronal genes are targets of RBFOX2 and RBFOX1 for alternative splicing, we were able to differentiate all of the RBFOX knockout iPSC lines into mature cortical neurons, demonstrating that the proteins are dispensible for neuronal differentiation. CNS-specific RBFOX2 and/or RBFOX1 knockout mice were viable and had morphologically normal cortical layers, which supports our observation^22^,^30^. However, there was a significant increase in proliferation at around 7-weeks of neural differentiation in RBFOX2 KO neurons from three independently-derived clones, despite normal cell cycle in iPSCs (Fig. 4C, Supplementary Figure 3C). This suggests that RBFOX2 may play an important role in the decision between self-renewal and differentiation during neural development. We found increased inclusion of the pro-proliferative exon 12 of *NUMB* in RBFOX2 KO neurons, which may provide an underlying mechanism for this proliferation phenotype, although we did not directly test this hypothesis. We measured mRNA levels of neural markers in mature RBFOX2 KO neurons to determine whether this increase in proliferation affects neural cell-fate. While most neural markers remained unchanged, we found that *TBR1* and *TBR2* were significantly upregulated in 10-week-old RBFOX2 knockout neurons. *PAX6, TBR2*, and *TBR1* are expressed sequentially during glutamatergic neocortical development^25^. With no change in *PAX6* level and significant increases in both *TBR2* and *TBR1* levels in RBFOX2 knockout neurons, we hypothesized that there was an over-proliferation in the *TBR2*^+^ intermediate progenitor population, leading to an increase in the post-mitotic *TBR1*^+^ neurons. However, the percentage of TBR1^+^ neurons was not increased in RBFOX2 KO neuron cultures. Many TBR1^+^ neurons reside in multi-layered hubs, however, that are hard to image and quantitate using microscopy (Supplementary Figure 9). Perhaps there are increased numbers of such hubs in RBFOX2 KO cultures compared to controls that may account for the expression differences.

We also sought to verify previously reported gene expression changes that were observed in RBFOX1 knockdown human neural precursor cells^26^. Most of the genes we analysed were not altered in RBFOX1 or RBFOX2 KO neurons. One of the genes analyzed, *FRZB*, was reported to be downregulated in RBFOX1 knockdown human neural progenitor cells^26^. While we did not see a change in *FRZB* in RBFOX1 knockout neurons, we observed a significant increase in *FRZB* in RBFOX2 knockout neurons (Fig. 5F, Supplementary Figure 4F). FRZB protein is a Wnt signaling antagonist that competes with FRIZZLED receptors for Wnt-binding^27^,^28^. Therefore, we quantified *AXIN2* expression. *AXIN2* is a downstream target of the canonical Wnt/β-catenin pathway. *AXIN2* expression was not different between RBFOX2 KO and control neurons, suggesting that the increased *FRZB* expression was not causing attenuation of the canonical Wnt/β-catenin pathway (Fig. 5F, Supplementary Figure 4F). It is possible that despite increased *FRZB* mRNA, FRZB protein was not increased. It is also possible that non-canonical Wnt pathways^34^,^35^, may be altered in RBFOX2 KO neurons.

Our previously published transcriptome data shows that the fragments per kilobase of transcript per million mapped reads (FPKM) values for RBFOX1, RBFOX2, and RBFOX3 are 16.5, 50.2, 5.8, respectively, in AS neurons and 16.5, 63.9, 6.8, respectively, in normal neurons^36^. A similar trend in gene expression is evident from the Brainspan data (www.brainspan.org)^31^,^32^. The fact that RBFOX2 is much more abundant than RBFOX1 in neurons at the developmental stage we assessed may explain why more severe phenotypes were observed in the absence of RBFOX2. Because of the highly conserved RNA-binding-motif between RBFOX1 and RBFOX2, we expected that they functionally compensate each other to a certain extent. However, the RBFOX1/2 dKO neurons presented gene expression changes consistent with RBFOX2 KO neurons, and showed similar splicing changes to RBFOX2 (Fig. 3C, Supplementary Figure 2G). This observation supports our notion that RBFOX2, the most abundant RBFOX paralog, plays a larger role than the other RBFOX proteins in these neurons. It is curious that the RBFOX1/2 dKO neurons have a slightly milder phenotype compared to RBFOX2 KO neurons. However, the most apparent phenotype in the RBFOX2 KO neurons is the increased proliferation in immature neural cultures. Perhaps the loss of RBFOX1 somehow counteracts this increased proliferation.

In conclusion, RBFOX1 and RBFOX2 are not required for processing of *SNURF/SNRPN* lncRNA, despite the fact that the lncRNA is highly bound by the RBFOX proteins. The absence of RBFOX1 did not overtly affect expression or splicing for genes assayed here and did not present observable phenotypes in *in vitro* derived human neurons, although genomewide analyses of splicing in human RBFOX1 KO neurons would likely reveal RBFOX1-specific targets. On the other hand, the absence of RBFOX2 led to a proliferation phenotype in immature neural cultures and altered the expression of some genes that are important for glutamatergic neocortical development. These data support the idea that RBFOX1 and RBFOX2 are not completely functionally redundant in early human neural development^30^.

## Material and Methods

### iPSC Culture

iPSCs were maintained as described before^4^. Briefly, iPSCs were grown on irradiated mouse embryonic fibroblasts and fed daily with conventional hESC medium consisting of DMEM-F12 supplemented with knock-out serum replacer, nonessential amino acids, L-glutamine, β-mercaptoethanol, and basic FGF. iPSCs were cultured in a humid incubator at 37 °C with 5% CO_2_ and passaged approximately once a week manually.

### Neural Differentiation

iPSC-derived neuronal culture were generated using either embryoid-body based^37^ or monolayer^38^ differentiation protocol with some modifications as previously reported^4^,^36^. Briefly, iPSC colonies were manually cut and lifted when generating embryoid bodies for the embryoid-body protocol. After three weeks of neural differentiation using either protocol, neural progenitors were plated on tissue culture plates coated with poly-ornithine/laminin. The neural differentiation medium consisted of Neurobasal Medium, B-27 supplement, nonessential amino acids, and L-glutamine, and was supplemented with 1 μM ascorbic acid, 200 μM cyclic adenosine monophosphate, 10 ng/mL brain-derived neurotrophic factor, and 10 ng/mL glial-derived neurotrophic factor. Unless otherwise specified, all experiments were conducted on neural cultures that were at least 10 weeks old.

### Lentiviral Production, Transduction, and Clone-screening

sgRNAs were designed using web-based CRISPR design tool (http://crispr.mit.edu) and cloned into lentiCRISPR (Addgene Plasmid 49535 and 52961) and lentiGuidePuro (Addgene Plasmid 52963) as instructed^19^,^39^. lentiviral particles were made by transfecting 293FT cells with 2^nd^ generation packaging systems using lipofectamine 2000 (Life Technologies). iPSCs were singlized using Accutase (Millipore) and transduced with lentivirus in suspension in the presence of 8 μg/mL polybrene for two hours. The iPSCs/lentivirus mixture were diluted 1:1 in hESC medium and plated on DR4 MEF feeders at a low density, supplemented with 10 μM ROCK inhibitor, Y-27632, overnight. Attached cells were cultured in hESC medium for an additional 72 hours before drug selection using puromycin at 0.5 μg/mL during the first week and at 1 μg/mL during the second week. Puromycin-resistant iPSC colonies were individually picked into a new feeder well and screened for indels by sequencing genomic DNA.

### RNA Isolation and Reverse Transcription (RT)

RNA samples were isolated using RNA-Bee (Tel-Test Inc.), DNase-treated with Amplification Grade DNaseI (Invitrogen) at 37 °C for 45 minute, and converted into cDNA using High-Capacity cDNA Reverse Transcription Kit (Applied Biosystems) according to the manufacturer’s instructions.

### RT- PCR Quantification for Splicing Events

Splicing events were quantified from conventional (non-quantitative) RT-PCR experiments using ImageJ software. Conventional RT-PCR was carried out as previously described^4^, and gel images were captured. Image color was inverted, and image background was subtracted at rolling ball radius of 50 pixels with light background. Image color was inverted again and integrated densities were measured using equal-sized boxed-in areas. For each gel picture, remaining background signal was taken from a equal-sized blank spot and subtracted by hand. Negative values can occur at this step when band intensity is not significant when compared to background and are therefore adjusted to zero. Ratios of exon-inclusion over exon-exclusion events were calculated for each sample and were normalized to un-manipulated controls.

### Quantitative Reverse Transcription PCR

All RT-qPCR assays were performed in biological triplicate from independent cultures, each with two technical replicates. Expression levels of target genes were measured using Taqman gene expression assays (Applied Biosystems) following the manufacturer’s protocol. In the case of *sno-lncRNAs*, gene expression levels were measured using published primers^21^ in combination with SYBR Green PCR master mix (Applied Biosystems). All genes were normalized to *GAPDH*. Relative quantity (RQ) value was calculated as 2^^−ΔΔCt^ using un-manipulated AS or normal cell lines as the calibrator sample.

Statistical analysis was carried out using Prism software (GraphPad). For each condition shown, averaged RQ values from biological triplicates were calculated and the resulting standard error of the mean was reported in the error bars. For each gene, averaged RQ values for each sample were compared to that of the un-manipulated control of the same genotype (AS or normal) and the significance for each un-manipulated vs KO pair was calculated using the one-way ANOVA with the Bonferroni post-test.

### Western Blotting

Cells were lysed on ice using RIPA buffer (150 mM NaCl; 50 mM Tris, pH 8.0; 1% TritonX-100; 0.5% Sodium Deoxycholate; 0.1% SDS) supplemented with proteinase inhibitor II. Protein concentrations were measured using Peirce BCA Protein Assay Kit (Thermo Scientific). Total cell lysates were resolved on 10% handcast SDS-PAGE gels and transferred to nitrocellulose or polyvinylidene difluoride membranes using wet/tank blotting system (Bio-Rad). The membranes were blocked in 5% nonfat dry milk in TBST (0.5% Tween-20 in TBS containing 20 mM Tris and 500 mM NaCl). The following primary antibodies and concentrations were used: mouse anti-GAPDH (1:3000, MAB374, Millipore), rabbit anti-RBFOX2 (1:2000, A300–864 A, Bethyl), rabbit anti-RBFOX1 (1:1000, ab83574, Abcam). After washing with TBST three times, membranes were blotted with corresponding secondary antibodies conjugated with horseradish peroxidase. Membranes were washed and then developed in Immobilon Western Chemiluminescent HRP Substrate (Millipore), visualized using ChemiDoc Touch (Bio-Rad). All western blot results were replicated in at least two biological replicates.

Protein levels were quantified using ImageJ software. Image background was subtracted at rolling ball radius of 50 pixels with light background. Image color was inverted and integrated densities were measured using equal-sized boxed-in areas. For each blot, remaining background signal was taken from a equal-sized blank spot and subtracted by hand. Negative values can occur at this step when protein level is not significant when compared to background and are therefore adjusted to zero. Protein level for each sample was normalized to its respective GAPDH level.

### Cross-linking Immunoprecipitation (CLIP)

CLIP experiments are carried out according to published protocols^9^,^40^. Briefly, RNA-binding proteins are cross-linked to RNA transcripts by UV irradiation at 400 mJ/cm^2^. Irradiated cells are lysed and subjected to DNase treatment. RBFOX1 and RBFOX2 bound transcripts are magnetically pulled down using Protein A Dynabeads (Dynal), which are pre-conjugated to rabbit anti-RBFOX1 (Abcam) and rabbit anti-RBFOX2 (Bethyl,^9^) respectively. An antibody against rabbit IgG is used to assay for non-specific binding. After purification steps, transcripts are released by proteinase K and isolated as described before. RNA products are reverse-transcribed followed by conventional PCR analysis.

### Immunocytochemistry

Immunocytochemistry was carried out as previously described^4^. The following antibodies and concentrations were used: rabbit anti-RBFOX2 (1:250, A300–864 A, Bethyl), mouse anti-OCT3/4 (1:100, sc-5279, Santa Cruz Biotechnology), rabbit anti-Activated-Caspase3 (1:400, #9661, Cell Signaling Technology), mouse anti-Ki67 (1:100, M7240, Dako), rabbit anti-TBR1 (1:500, ab31940, Abcam). AlexaFluor 488 and 594 fluorochrome conjugated secondary antibodies (Life Technologies) were used at 1:500. Nuclei were counterstained with DAPI and coverslips were mounted on slides with Vectashield (Vector Laboratories). Slides were imaged using a Zeiss Axiovision microscope or a Zeiss LSM 780 confocal microscope.

Cell counting was carried out in a double-blind fashion in biological triplicates on independent cultures. For each coverslip, eight pictures containing a total of at least 1000 DAP^+^ cells were counted (a minimum of 3000 cells per cell line). Statistical analysis was carried out using Prism software (GraphPad). Total number of Ki67^+^ or TBR1^+^ cells on each coverslip was normalized to that of DAPI^+^ cells. Averaged ratios of Ki67^+^/DAPI^+^ cells were calculated from biological triplicates and the resulting standard deviations were used for the error bars. For each cell line, averaged ratio was compared to that of respective un-manipulated control and significance was calculated using one-way ANOVA with Bonferroni post-test.

### Cell Death and Cell Cycle Flow Cytometry

Cell death analysis was done using Dead Cell Apoptosis Kit with Annexin V Alexa Fluor 488 and propidium iodide (Molecular Probes). Seven days after splitting, iPSCs were singlized using Accutase (Millipore). Staining was carried out according to the manufacturer’s protocol. All experiments were run in triplicates using independent cultures. Negative control with no Annexin V and propidium iodide staining, Annixin V only control, and propidium iodide only control were included using corresponding cell lines to set up gates. Flow cytometry analysis was carried out by UCONN Health Flow Cytometry Core, using a MACSQuant Analyzer 10.

Cell cycle analysis was done using Click-iT Plus EdU Alexa Fluor 647 Flow Cytometry Assay Kit (Molecular Probes). Four days after splitting, iPSCs were synchronized using 0.2 μM of nocodazole for 18 hours. The cells were then released into cell cycle for 3.5 hours in hESC medium, followed by 10 μM EdU labeling for 30 minutes. Cells were then washed and singlized using TrypLE Express (Gibco). Staining was carried out according to the manufacturer’s protocol. All experiments were run in duplicates or triplicates using independent cultures. In addition to EdU-labeling and propidium iodide, iPSCs were also stained with mouse anti-OCT3/4 (1:100, sc-5279, Santa Cruz Biotechnology) in combination with goat anti-mouse AlexaFluor 488 fluorochrome conjugated secondary antibody (1:200, Life Technologies) for pluripotency. Negative control with no EdU, Oct3/4, and propidium iodide staining, EdU-labeling only control, Oct3/4 only control and propidium iodide only control were included using corresponding cell lines to set up gates. AlexaFluor 488 fluorochrome conjugated secondary antibody was present in all four controls. Flow cytometry analysis was carried out by UCONN Health Flow Cytometry Core, using a Becton-Dickinson LSR II Flow Cytometer.

Statistical analysis was carried out using Prism software (GraphPad). Averaged percentage from biological replicates was calculated and the resulting standard deviation was used for error bar. For each group, averaged percentage for each sample was compared to that of their respective un-manipulated control and significance was calculated using one-way ANOVA with Bonferroni post-test.

### RNA FISH

RNA fluorescence *in situ* hybridization was carried out as previously described^41^. *SNORD116* probes were made from BAC RP11–186C7 (BACPAC Resources Center). Slides were imaged using a Zeiss LSM 780 confocal microscope.

## Additional Information

**How to cite this article**: Chen, P.-F. *et al.* RBFOX1 and RBFOX2 are dispensable in iPSCs and iPSC-derived neurons and do not contribute to neural-specific paternal *UBE3A* silencing. *Sci. Rep.* **6**, 25368; doi: 10.1038/srep25368 (2016).

## Supplementary Material

Supplementary Information
