# Supplementary material for: RBFOX1 and RBFOX2 are dispensable in iPSCs and iPSC-derived neurons and do not contribute to neural-specific paternal UBE3A silencing
Source: Sci Rep. 2016 May 5;6:25368. doi: 10.1038/srep25368 (PMC4857170; doi:10.1038/srep25368)
Supplement: Supplementary Information [file srep25368-s1.docx]

**Supplementary information for:**

**RBFOX1 and RBFOX2 are dispensable in iPSCs and iPSC-derived neurons and do not contribute to neural-specific paternal *UBE3A* silencing**

Pin-Fang Chen^1^, Jack S. Hsiao^1^, Carissa L. Sirois^2^ and Stormy J. Chamberlain^1,3,*^

^1^ Department of Genetics and Genome Sciences, University of Connecticut Health Center, Farmington, Connecticut, 06030, USA

^2^ Department of Neuroscience, University of Connecticut Health Center, Farmington, Connecticut, 06030, USA

^3^ University of Connecticut Stem Cell Institute, University of Connecticut Health Center, Farmington, Connecticut, 06030, USA


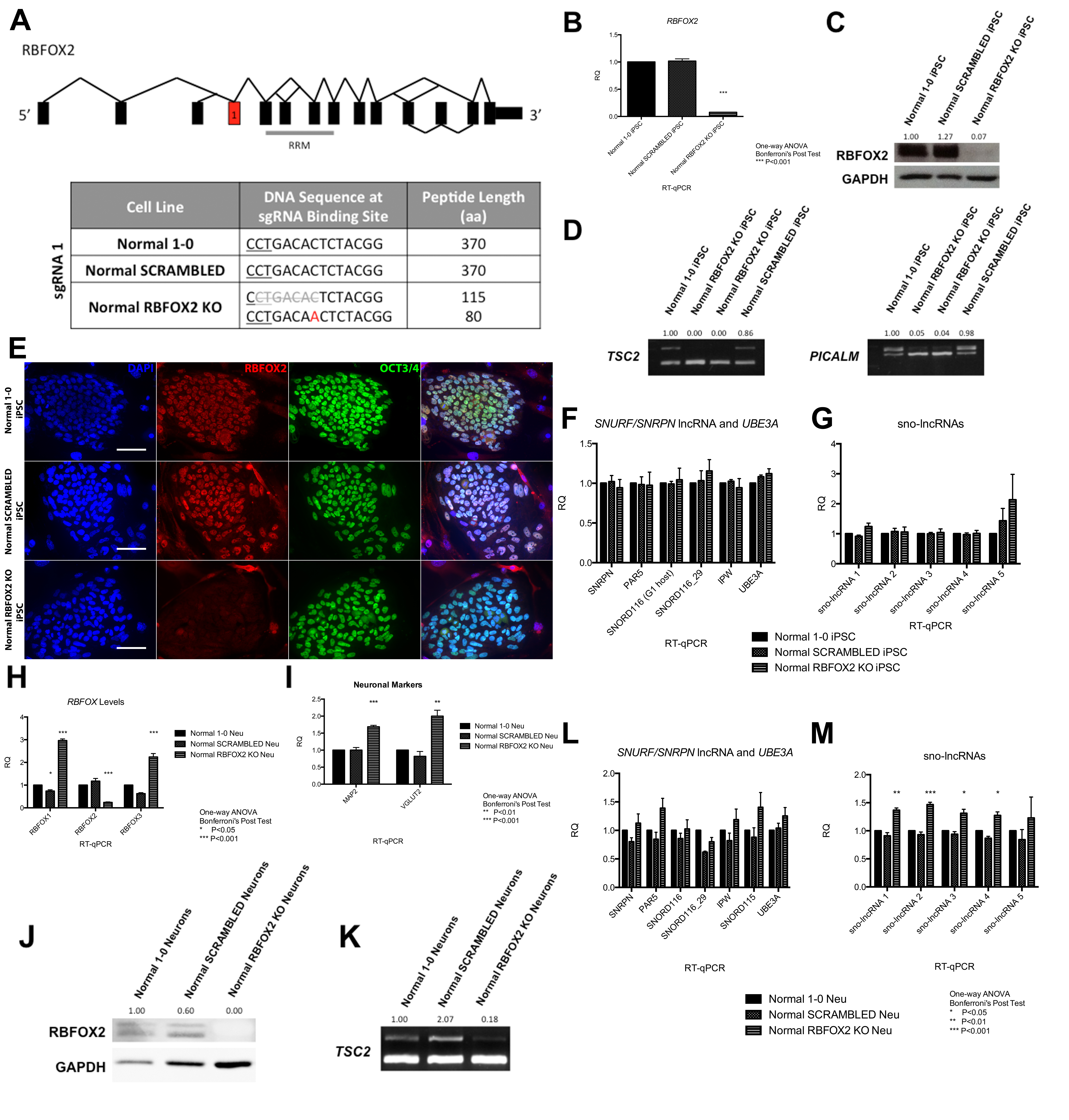


**Supplementary Figure 1.** Lentiviral CRISPR/Cas9-mediated RBFOX2 KO in Normal iPSCs. The expression of *SNURF/SNRPN* lncRNA was not altered in RBFOX KO AS iPSCs and iPSC-derived neurons. **A)** Schematic of *RBFOX2* splicing pattern according to alternative splicing graph from Swiss Institute of Bioinformatics on UCSC genome browser. Targeted exon is colored in red. gDNA sequencing shows frameshift indels leading to premature stop codon in the RBFOX2 KO. **B, C, and E)** RT-qPCR, western blot, and immunocytochemistry showing *RBFOX2*/RBFOX2 expression in the RBFOX2 KO iPSCs. Scale bar, 100μm. Relative protein levels to un-manipulated normal iPSCs are shown in numbers. **D)** Splicing changes in *TSC2* and *PICALM* showing functional loss of RBFOX2. Levels of exon-inclusion over exon-exclusion events relative to un-manipulated normal iPSCs are shown in numbers. **F and G)** RT-qPCR to quantify transcripts from the *SNURF/SNRPN* lncRNA, including *SNORD116* and *sno-lncRNAs* in RBFOX2 KO iPSCs. **H)** RT-qPCR showing reduced *RBFOX2* expression in RBFOX2 KO neurons. **I)** Increased *MAP2* and *VGLUT2* expression corresponded with increased *RBFOX1* expression in H. **J)** Western blot showing loss of RBFOX2 in KO neurons. Relative protein levels to un-manipulated normal iPSCs are shown in numbers. **K)** Splicing changes in *TSC2* showing functional loss of RBFOX2 in KO neurons. Levels of exon-inclusion over exon-exclusion events relative to un-manipulated normal iPSCs are shown in numbers. **L and M)** RT-qPCR to quantify transcripts from *SNURF/SNRPN* lncRNA, including *SNORD116* and *sno-lncRNAs*, in RBFOX KO neurons.


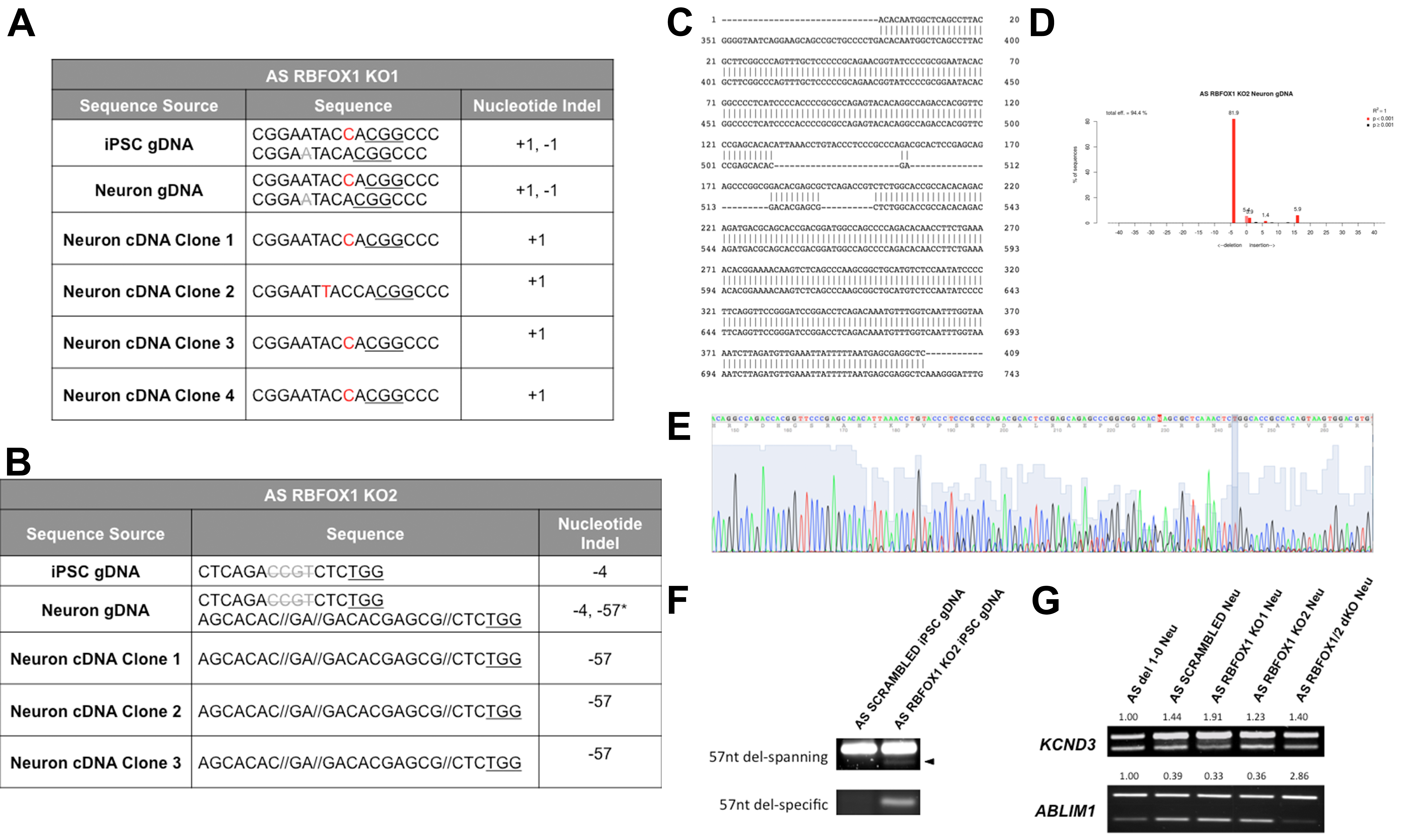


**Supplementary Figure 2.** Sequencing analysis for *RBFOX1* mutations in AS RBFOX1 KO iPSCs and neurons. **A and B)** gDNA sequencing in iPSCs and neurons as well as cDNA sequencing by pBluescript cloning of PCR products in AS RBFOX1 KO1 and KO2. **C)** Sequencing alignment between representative AS RBFOX1 KO2 pBluecript cloning product and control wild type sequence, showing a complex 57 nucleotide deletion. **D)** The 57 nucleotide deletion in C was not picked up by TIDE in AS RBFOX1 KO2 neuronal gDNA due to the software’s size limitation on indels. **E)** AS RBFOX1 KO2 neuronal gDNA trace file showing mutations that can be resolved into the 57 nucleotide deletion by hand. **F)** By conventional PCR, 3% agarose gel image showed a small population of AS RBFOX1 KO2 iPSCs harbors the 57 nucleotide deletion (arrowhead), which was better detected using primers specific to the deletion. **G)** Splicing changes in *KCND3* and *ABLIM1* showing functional loss of RBFOX1 in AS RBFOX1 KO and RBFOX1/2 dKO neurons. Numbers indicate levels of exon-inclusion over exon-exclusion events relative to un-manipulated AS del 1-0 neurons. Differences in these spliced isoform ratio are present in the scrambled neurons, which has lower RBFOX1 expression than the control neurons (Figure 3A).


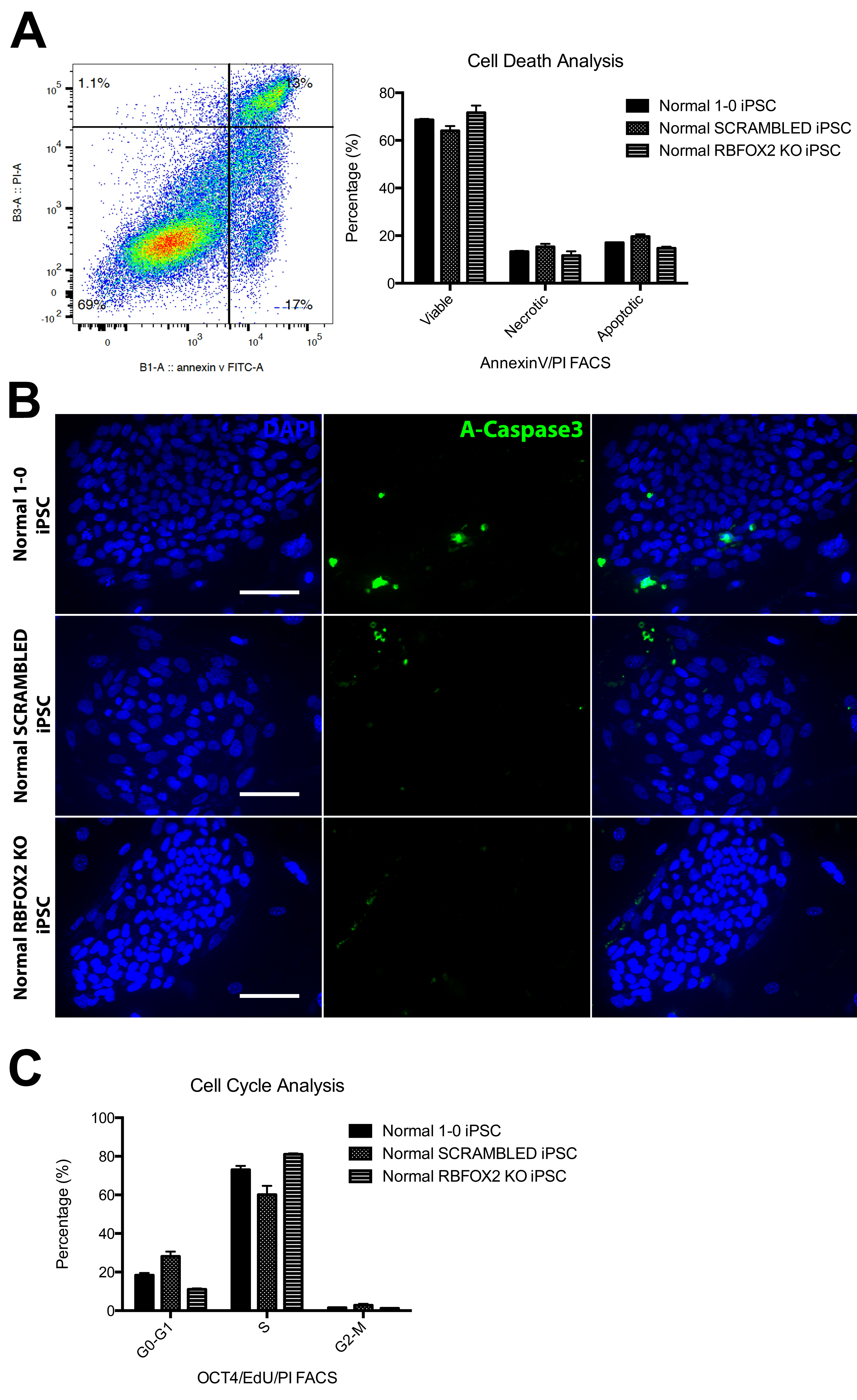


**Supplementary Figure 3.** Cell death and cell cycle analysis in RBFOX2 KO normal iPSCs. **A)** Flow cytometry analysis using Annexin V and propidium iodide, to identify viable, necrotic, or apoptotic cells. **B)** Apoptotic cells labeled with activated caspase-3 (green). Scale bar, 100μm. **C)** Flow cytometry was used to measure cell cycle in synchronized iPSCs. Cells were labeled with EdU during S-phase, and then stained with propidium iodide.


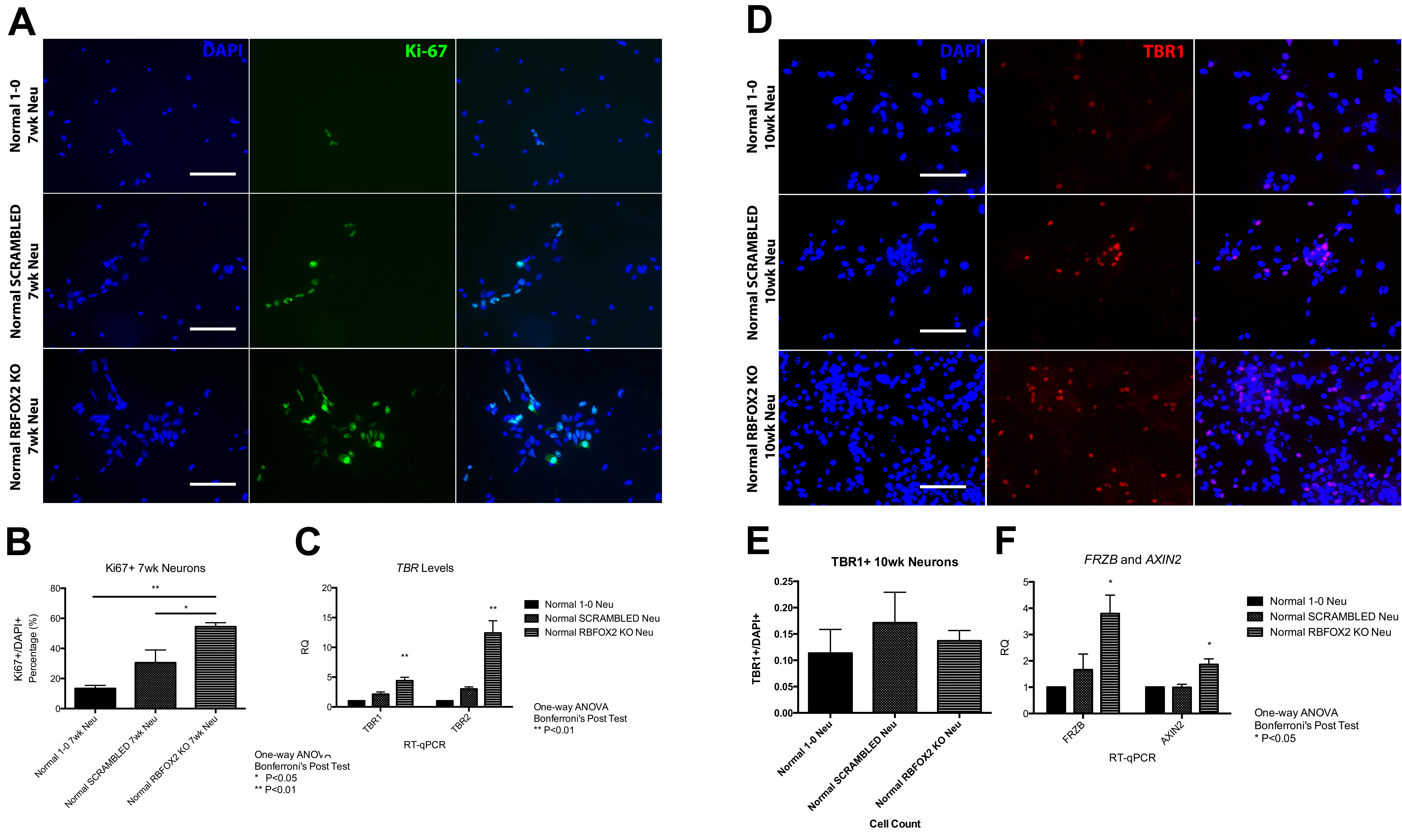


**Supplementary Figure 4.** 7-week-old normal RBFOX2 KO neurons showed increased proliferation and 10-week-old normal RBFOX2 KO neurons showed increased *TBR1*, *TBR2*, and *FRZB* expression. **A)** Proliferating cells in 7-week-old neural culture labeled with antibody against Ki67 (green). Scale bar, 100μm. **B)** Quantification of Ki67+ cells in A. **C)** RT-qPCR showing *TBR1* and *TBR2* expression in 10-week-old RBFOX2 KO neurons. **D)** 10-week-old RBFOX2 KO neurons labeled with antibody against TBR1 (red). Scale bar, 100μm. **E)** Quantification of TBR1+ cells in D. **F)** RT-qPCR showing *FRZB* and *AXIN2* expression in 10-week-old RBFOX2 KO neurons.


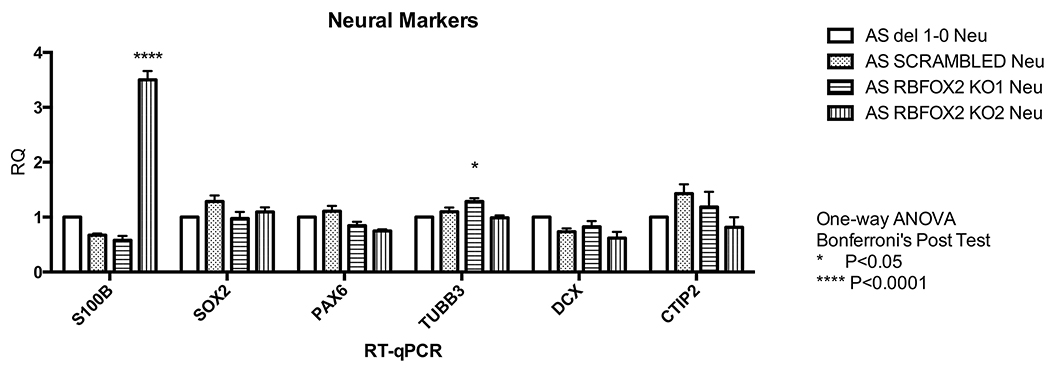


**Supplementary Figure 5.** RT-qPCR for neural markers in AS neurons. Altered S100B levels are likely caused by batch-to-batch differences in neural differentiation.


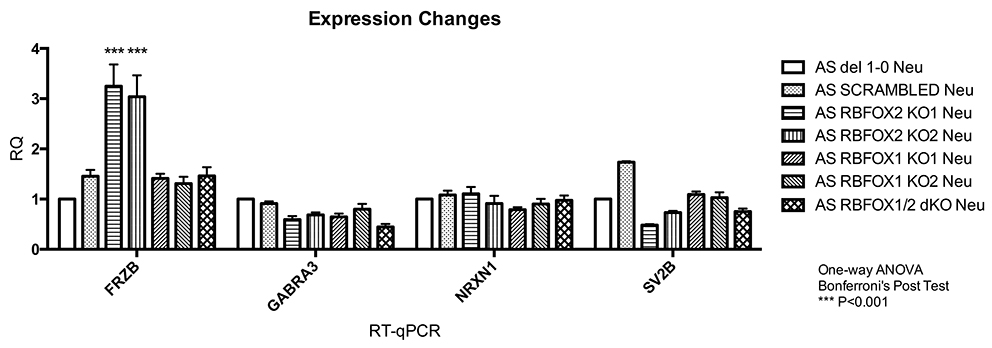


**Supplementary Figure 6.** RT-qPCR for genes previously shown to change in RBFOX1 knockdown human neural progenitor cells.


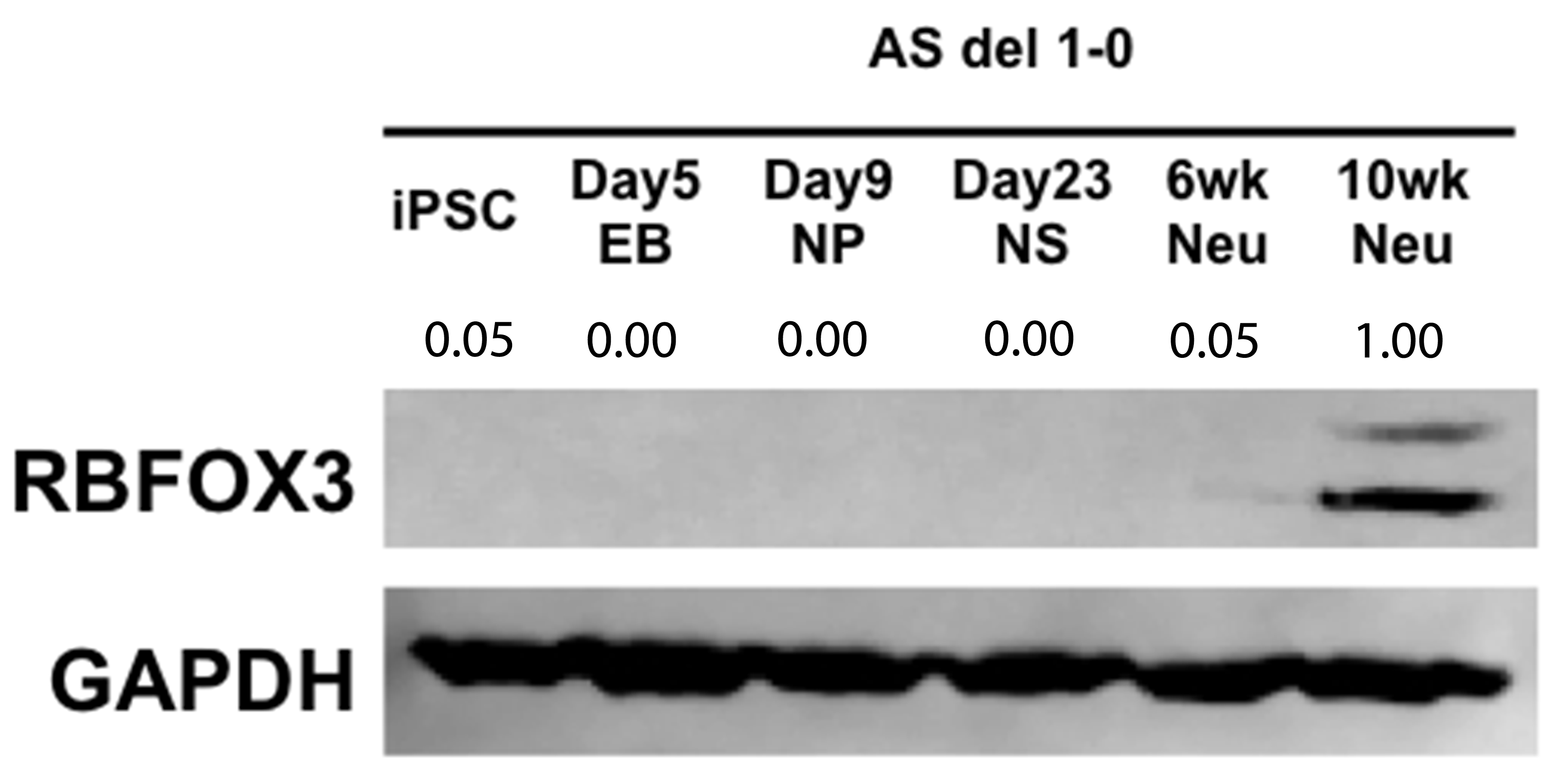


**A**

**B**

**
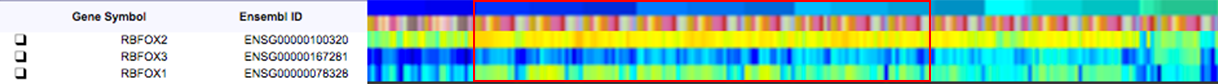
**

**C**

**
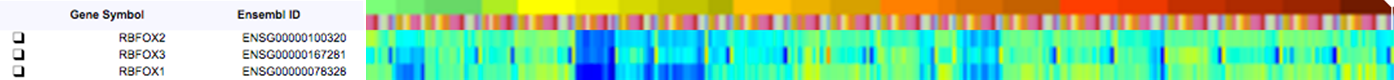
**

**Supplementary Figure 7. A.** Western blot showing RBFOX3 protein expression is only detectable in 10-week-old neuronal culture during neural differentiation. Relative protein levels to 10-week-old neurons are shown in numbers. **B.** Developmental transcriptome data for RBFOX1, RBFOX2, and RBFOX3 during prenatal ages 8 -37 weeks post-conception from Brainspan: Atlas of the human developing brain (www.brainspan.org)(1,2). Post-conception weeks 11-16 are most representative of our iPSC-derived neurons and are boxed in red. **C.** Developmental transcriptome data for RBFOX1, RBFOX2, and RBFOX3 during postnatal ages 4 months to 40 years from the same source. For **B** and **C,** blue shades represent lowest expression levels and yellow/orange shades represent the highest expression levels. The top two rows in each figure represent the developmental stage and the brain region, respectively. Samples are ordered by age from youngest to oldest.


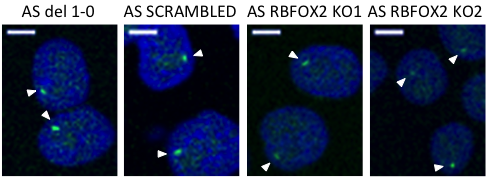


**Supplementary Figure 8.** RNA fluorescence in situ hybridization showing that localization of *SNORD116* transcripts (white arrow heads, labeled in green) is not altered in the absence of RBFOX2 protein. Scale bar, 5μm.


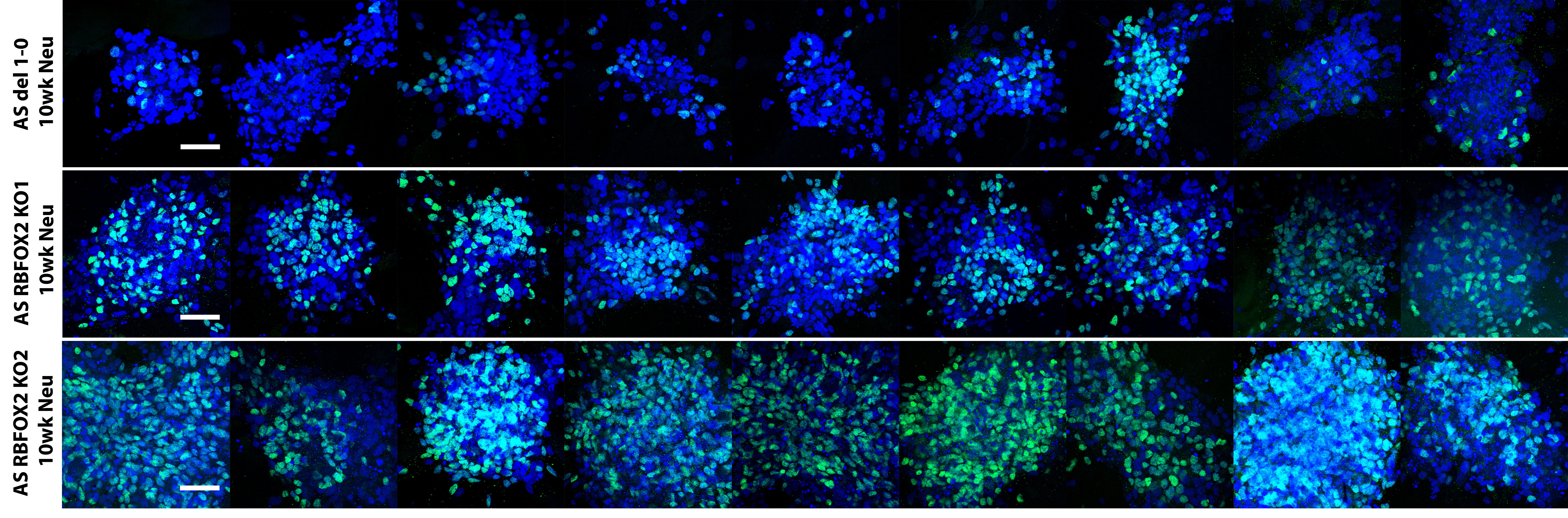


**Supplementary Figure 9.** Representative confocal microscopic images of TBR1^+^ cells (green) in multilayered hubs in 10-week-old neuronal culture from three independent experiments. Scale bar, 50μm.

**Supplementary Table 10. List of primers.**

**Primers for conventional PCR**

| **Gene** | **Forward Primer** | **Reverse Primer** | **Ref.** |
| --- | --- | --- | --- |
| ***RBFOX1*** | GACACAATGGCTCAGCCTTAC | CGGAACCTGAAGGGGATATT |  |
| ***RBFOX2*** | GGTGGAAAAGAAAGCTGTGG | GTGGTGGAAATGGGATGGTA |  |
| ***UBE3A-ATS*** | AAGGCCTGGAATCTGATCCT | CCTAGATTTTAAATAGACAATCCAAAG | (3) |
| ***PICALM*** | ATTCCATATCCTATCATGCCTGT | AATTGGAGTCAACCAGGTGAA | (4) |
| ***TSC2*** | CGGTCCAATGTCCTCTTGTC | TCCAGGTGGAGGTTTTTCAG | (4) |
| ***NUMB* (exon 12)** | ATCTGCTCCGATGACCAAAC | GAGAGGCAGCACCAGAAGAT |  |
| ***KCND3*** | AAGACCACCTCACTCATCG | TTCTTGTGGATGGGTAGTTC |  |
| ***ABLIM1*** | CTCCATCAACTCCCCTGTGT | TAGATGGGTGGCTTTCGGTA |  |

**CLIP primers**

| **Gene** | **Forward Primer** | **Reverse Primer** |
| --- | --- | --- |
| ***SNRPN*** | CCAACCTCTGGCATAAATGG | GACCTTCAGCCATCCAAAGA |
| ***PAR5*** | AGGTGCTTTTGCTTTGCCTA | TCTCTGAACCCCAACAGCTT |
| ***SNORD108*** | TGAGGTCCAGCCTTGCTAAT | CCACAATTCAAACCATGCAA |
| ***SNRPN* (RBFOX2 cluster)** | TAATTGGCCTCTTGGGACTG | TGACCATTCCCTTCTCCTGT |
| ***SNORD116*** | TCATAGTGCAGCCAGGACAG | TTTTCCTTGCATTGGACACA |
| ***IPW*** | TCTTCTGCCTCCTGTCTCGT | TCCCATCACCACAGTGAAAA |
| ***PAR1*** | AGCTGCCCACACCCATATAC | GTGGGGGCTCACACATAACT |
| ***SNORD115*** | TGGACACATGTCCTCCTCCT | TGGGATCTCAGCATCCTCTT |
| ***UBE3A-ATS(9)*** | GCCTTTGGAGACAACTTCCA | AGCCCTGAATTCTCACAGAAA |
| ***UBE3A-ATS(3)*** | AGTCTTGGGCTTCCTTTGGT | GAGGAACCATTGCAGCTGAT |
| ***UBE3A-ATS(1)*** | ACTTGGGCTCTACTCGCAAA | TGGCAAGTGAGTGTGCCTAA |

**Taqman RT-qPCR**

| **Gene** | **Assay ID** |
| --- | --- |
| ***RBFOX1*** | Hs01125659_m1 |
| ***RBFOX2*** | Hs00204814_m1 |
| ***RBFOX3*** | Hs01370653_m1 |
| ***SNRPN*** | Hs00256090_m1 |
| ***PAR5*** | Hs03453940_s1 |
| ***SNORD116*** | Hs03454084_m1 |
| ***SNORD116-29*** | Hs03300097_s1 |
| ***IPW*** | Hs03455409_s1 |
| ***SNORD115*** | Hs03454279_m1 |
| ***UBE3A*** | Hs00166580_m1 |
| ***SOX2*** | Hs01053049_s1 |
| ***PAX6*** | Hs00240871_m1 |
| ***DCX*** | Hs00167057_m1 |
| ***TUBB3*** | Hs00964962_g1 |
| ***CTIP2 (BCL11B)*** | Hs01102259_m1 |
| ***S100B*** | Hs00902901_m1 |
| ***MAP2*** | Hs00258900_m1 |
| ***VGLUT2 (SLC17A6)*** | Hs00220439_m1 |
| ***GAD1*** | Hs01065893_m1 |
| ***TBR1*** | Hs00232429_m1 |
| ***TBR2 (EOMES)*** | Hs00172872_m1 |
| ***SV2B*** | Hs00208178_m1 |
| ***NRXN1*** | Hs00985123_m1 |
| ***GABRA3*** | Hs00968132_m1 |
| ***FRZB*** | Hs00173503_m1 |
| ***AXIN2*** | Hs00610344_m1 |

**SYBR Green RT-qPCR**

| **Gene** | **Forward Primer** | **Reverse Primer** | **Ref.** |
| --- | --- | --- | --- |
| ***GAPDH*** | TACTAGCGGTTTTACGGGCG | TCGAACAGGAGGAGCAGAGAGCGA | (5) |
| ***sno-lncRNA 1*** | CTTGGCGTATTCATGGAGGT | ACCGGCTAAGTGAGCTGAAA | (6) |
| ***sno-lncRNA 2*** | TGTCCTTGACTCCTGGCTCT | ATGCCAGGTGATTGGAACTC | (6) |
| ***sno-lncRNA 3*** | CGTGCATCCCTATGTACGTG | CAATGCTACCTGGGAGGTGT | (6) |
| ***sno-lncRNA 4*** | GGCCAGAGACAGGCAGATAG | GTATCTCCGCAGCTCACACA | (6) |
| ***sno-lncRNA 5*** | GGAACCAGGGCATAGTGAGA | TTGGATTTGGATGTTGACCA | (6) |

1. Hawrylycz, M.J., Lein, E.S., Guillozet-Bongaarts, A.L., Shen, E.H., Ng, L., Miller, J.A., van de Lagemaat, L.N., Smith, K.A., Ebbert, A., Riley, Z.L. *et al.* (2012) An anatomically comprehensive atlas of the adult human brain transcriptome. *Nature*, **489**, 391-399.

2. Sunkin, S.M., Ng, L., Lau, C., Dolbeare, T., Gilbert, T.L., Thompson, C.L., Hawrylycz, M. and Dang, C. (2013) Allen Brain Atlas: an integrated spatio-temporal portal for exploring the central nervous system. *Nucleic Acids Res*, **41**, D996-D1008.

3. Runte, M., Huttenhofer, A., Gross, S., Kiefmann, M., Horsthemke, B. and Buiting, K. (2001) The IC-SNURF-SNRPN transcript serves as a host for multiple small nucleolar RNA species and as an antisense RNA for UBE3A. *Hum Mol Genet*, **10**, 2687-2700.

4. Yeo, G.W., Coufal, N.G., Liang, T.Y., Peng, G.E., Fu, X.D. and Gage, F.H. (2009) An RNA code for the FOX2 splicing regulator revealed by mapping RNA-protein interactions in stem cells. *Nat Struct Mol Biol*, **16**, 130-137.

5. Cruvinel, E., Budinetz, T., Germain, N., Chamberlain, S., Lalande, M. and Martins-Taylor, K. (2014) Reactivation of maternal SNORD116 cluster via SETDB1 knockdown in Prader-Willi syndrome iPSCs. *Hum Mol Genet*, **23**, 4674-4685.

6. Yin, Q.F., Yang, L., Zhang, Y., Xiang, J.F., Wu, Y.W., Carmichael, G.G. and Chen, L.L. (2012) Long noncoding RNAs with snoRNA ends. *Mol Cell*, **48**, 219-230.
